# Imaging features of fumarate hydratase-deficient renal cell carcinomas: a retrospective study

**DOI:** 10.1186/s40644-021-00392-9

**Published:** 2021-02-19

**Authors:** Ines Nikolovski, Maria I. Carlo, Ying-Bei Chen, Hebert Alberto Vargas

**Affiliations:** 1 Department of Radiology, Memorial Sloan Kettering Cancer Center, 300 E 66th St #1407, NY 10065 New York, USA; 2 Department of Medicine, Memorial Sloan Kettering Cancer Center, New York New York, USA; 3 Department of Pathology, Memorial Sloan Kettering Cancer Center, New York New York, USA

**Keywords:** FH-deficient, Hereditary leiomyomatosis and renal cell carcinoma syndrome, HLRCC

## Abstract

**Backgound:**

Fumarate hydratase-deficient renal cell carcinoma (FH-RCC) is a subtype of RCC that is increasingly recognized pathologically. The aim of this study was to evaluate the imaging features of FH-RCC on computed tomography (CT), magnetic resonance imaging (MRI), and fluorodeoxyglucose positron emission tomography (FDG PET), and to determine the pre-operative diagnostic potential of imaging.

**Methods:**

This single-site retrospective study included patients with histologically confirmed FH-RCC or with a renal cancer and known germline FH mutation; imaging of the renal mass before treatment with contrast-enhanced CT, contrast-enhanced MRI, or FDG PET/CT between October 2007 and May 2019. Clinical information, pathological data, and imaging features were analyzed and reported descriptively.

**Results:**

Sixteen patients with sixteen tumors were included (median age 46 years, interquartile range 38–53 years; 31 % female). Almost all tumors were unifocal (15/16, 94 %). Most tumors had infiltrative margins (14/16, 88 %); few were circumscribed (2/16, 12 %). A large cystic tumor component (> 75 % of tumor volume) was seen in 8/16 (50 %) of tumors. Involvement of renal sinus fat was seen in 13/16 (81 %) of tumors, involvement of the hilar collecting system in 8/16 (50 %), and renal vein tumor thrombus in 6/16 (38 %). All 12 tumors (100 %) imaged with MRI had heterogenous tumor enhancement and heterogenous T2 signal. Of those patients that had diffusion-weighted imaging, 11/11 (100 %) of tumors had diffusion restriction in the solid portions of the tumor. Of the patients who had PET, 3/3 (100 %) tumors showed high metabolic activity with mean maximum standardized uptake value (SUV_max_) of 16.4 (range 9.6–21.9). Patients presented with retroperitoneal nodal metastases in 69 % of cases and distant metastases in 75 %. Of those four patients without metastatic disease at presentation, three (75 %) developed metastases within 4 years of diagnosis.

**Conclusions:**

In our study, the majority of tumors (≥ 75 %) were unifocal, had an infiltrative margin, invaded the renal sinus fat, and presented with distant metastases. On MRI, most tumors had heterogenous T2 signal and diffusion restriction in their solid components. The small number of cases that had PET imaging showed high metabolic activity.

## Background

Fumarate hydratase deficient renal cell carcinoma (FH-RCC) is a rare subtype of RCC. FH-RCC usually occurs in the context of hereditary leiomyomatosis and renal cell carcinoma (HLRCC) syndrome, an autosomal dominant disorder characterized by uterine and cutaneous leiomyomas and increased predisposition to an aggressive form of RCC [1]. HLRCC is caused by heterozygous germline mutations in the FH gene located on chromosome 1, which encodes FH, a critical component of the Krebs cycle [2, 3]. The mutations impair oxidative phosphorylation and lead to the accumulation of oncometabolites [4]. Recent work by Shuch et al. has found that FH alterations are more common than previously suspected and are carried by approximately 1 in 1000 individuals, however the lifetime kidney cancer penetrance appears lower than previously described, with an estimate based on one definition of carrier frequency ranging from 3.6 to 11.9 % [5]. Since 2016, the World Health Organization classification of tumors of the urinary system has included HLRCC-associated RCC as a separate entity [6]. However, as opposed to HLRCC-associated RCC, less commonly, histologically indistinguishable tumors can also arise from biallelic somatic loss of FH, without the germline mutations denoting HLRCC syndrome.

FH-RCC has been described in the pathology literature as typically having FH-deficiency or 2-succino-cysteine (2SC) positive immunoreactivity on immunohistochemical (IHC) analysis, and can be distinguished histologically by papillary architecture with abundant eosinophilic cytoplasm, large nuclei, and very prominent nucleoli with perinucleolar clearing [7–9]. The imaging characteristics of FH-RCC, however, remain largely unknown, beyond the fact that these tumors are usually metastatic at presentation and typically solitary and unilateral, unlike other familial RCC syndromes that are characterized by bilateral multifocal tumors [10]. The lack of radiology literature available for FH-RCC is probably due to the fact that these tumors are uncommon compared with non-FH deficient RCC subtypes, and can be difficult to diagnose pathologically, requiring the pathologist to be vigilant when encountering renal masses with unusual or unclassified histologic features [11]. Knowledge of the clinical and pathologic characteristics of HLRCC has been expanding since it was first described twenty years ago, but it is still unknown whether there are any distinguishing imaging features that may allow for accurate preoperative diagnosis. Such imaging features have importance for both the individual patient and their extended family members given the often-aggressive nature of these tumors and the likelihood of a hereditary cancer syndrome. Imaging diagnosis may also assist the pathologist in ensuring confirmatory FH or 2-SC IHC analysis of the surgical specimen is performed, testing which is not routine in RCC.

The purpose of this study was to document the imaging features of FH-RCC on computed tomography (CT), magnetic resonance imaging (MRI), and fluorodeoxyglucose positron emission tomography CT (FDG PET/CT) in order that these may aid in the prospective identification of patients who may be at risk of HLRCC and a more aggressive disease course.

## Methods

 The Institutional Review Board approved this retrospective, Health Insurance Portability and Accountability Act-compliant single-site study and waived written informed consent. The inclusion criteria for the study were (i) patient with a renal mass and presence of FH germline mutation, or a renal mass with FH loss and/or 2-succino-cysteine (2SC) positive immunoreactivity on IHC analysis, (ii) imaging of the renal mass before treatment with contrast-enhanced CT, contrast-enhanced MRI or FDG PET/CT, (iii) imaging study available in Digital Imaging and Communications in Medicine (DICOM) format through our institution’s picture archiving and communications system (PACS) and, (iv) imaging study performed between date range of 10/01/2007 and 05/01/2019. Sixteen patients satisfied all the above inclusion criteria.

The CT and MRI studies were a combination of in-house and outside studies performed on multiple different scanners. All studies included post-contrast images, and the MRI studies included standard abdominal sequences, with variability in scanner and protocol not affecting the qualitative features that were analyzed. All imaging studies were performed prior to histologic evaluation and genetic testing and reviewed retrospectively by a single genitourinary radiologist (IN) with 5 years of post-fellowship experience. For all CT and MRI studies, the radiologist documented tumor size, location, focality, ill-defined or circumscribed margins, presence of large cystic tumor component (defined as greater than 75 % tumor volume involved by component with fluid density on CT or signal intensity on MRI), invasion of renal sinus fat and/or collecting system, presence of peritumoral vascularity, presence and heterogeneity of enhancement, renal vein invasion, retroperitoneal nodal metastases (defined as lymph node measuring greater than 1 cm short axis and/or suspicious morphologic features such as rounded margin and heterogeneity), and distant metastases. Additional modality-specific tumor features were also recorded, such as presence of calcifications on CT, and presence of intravoxel fat, T1 hyperintense hemorrhage, T2 signal heterogeneity, and diffusion restriction on MRI. For PET, the presence of tumor FDG avidity above liver background and mean maximum standardized uptake value (SUV_max_) were recorded.

## Results

### Clinical features

This study included 16 patients with 16 tumors. Patients ranged in age from 20 to 73 years (median 46 years, interquartile range 38–53 years); 11 patients were male, and 5 were female. Germline FH mutation was present in 13 patients indicating they had HLRCC (only 1 patient was aware of having HLRCC prior to presentation with FH-RCC), and the remaining 3 patients had somatic FH mutations only. The tumors were predominantly unilateral and solitary (15/16, 94 %), apart from one patient who had bilateral multifocal tumors, of which only the largest tumor was analyzed. There was no predilection for kidney laterality. Thirteen patients had a CT scan with contrast, 12 had MRI with contrast (of those, 11 had diffusion-weighted images available for review), and three had an FDG PET/CT study. Clinical and demographic characteristics, and the number of cases of each imaging modality, are presented in Table 1.

**Table 1:** Patient Demographics

Characteristics	n (%)
Total	16
Male	11 (69)
Female	5 (31)
Median age in years (Interquartile range)	46 (38–53)
Germline FH mutation (HLRCC)	13 (81)
Somatic FH mutation	3 (19)
CT with contrast	13
MRI with contrast	12
DWI	11
FDG PET/CT	3
Mean follow up time in months (range)	26 (9–60)

*FH* fumarate hydratase, *HLRCC* hereditary leiomyomatosis and renal cell carcinoma, *CT* computed tomography, *DWI* diffusion-weighted imaging, *FDG* *PET/CT* fluorodeoxyglucose positron emission tomography

### Imaging features

**Fig. 1:**
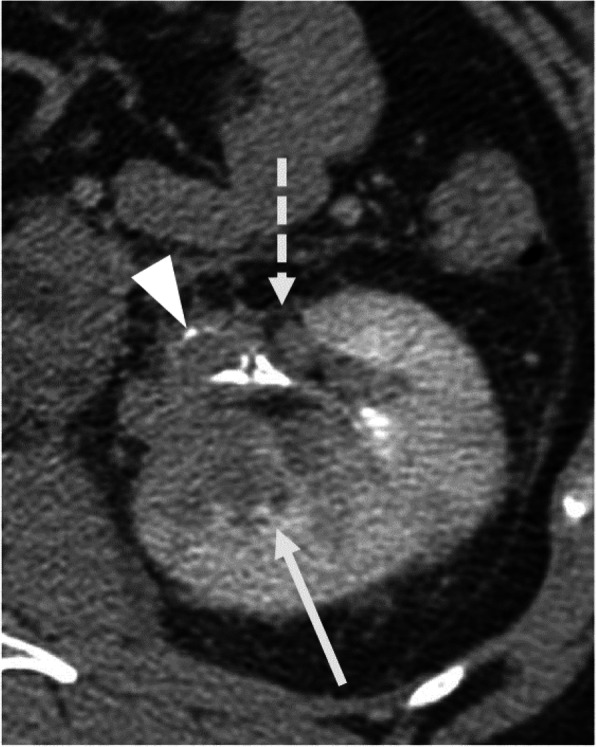
45-year-old female with metastatic fumarate hydratase-deficient renal cell carcinoma. Delayed phase post-contrast axial computed tomography showing endophytic renal mass with infiltrative margin (arrow), renal sinus fat invasion (dashed arrow), and hilar collecting system invasion (arrowhead)

**Fig. 2:**
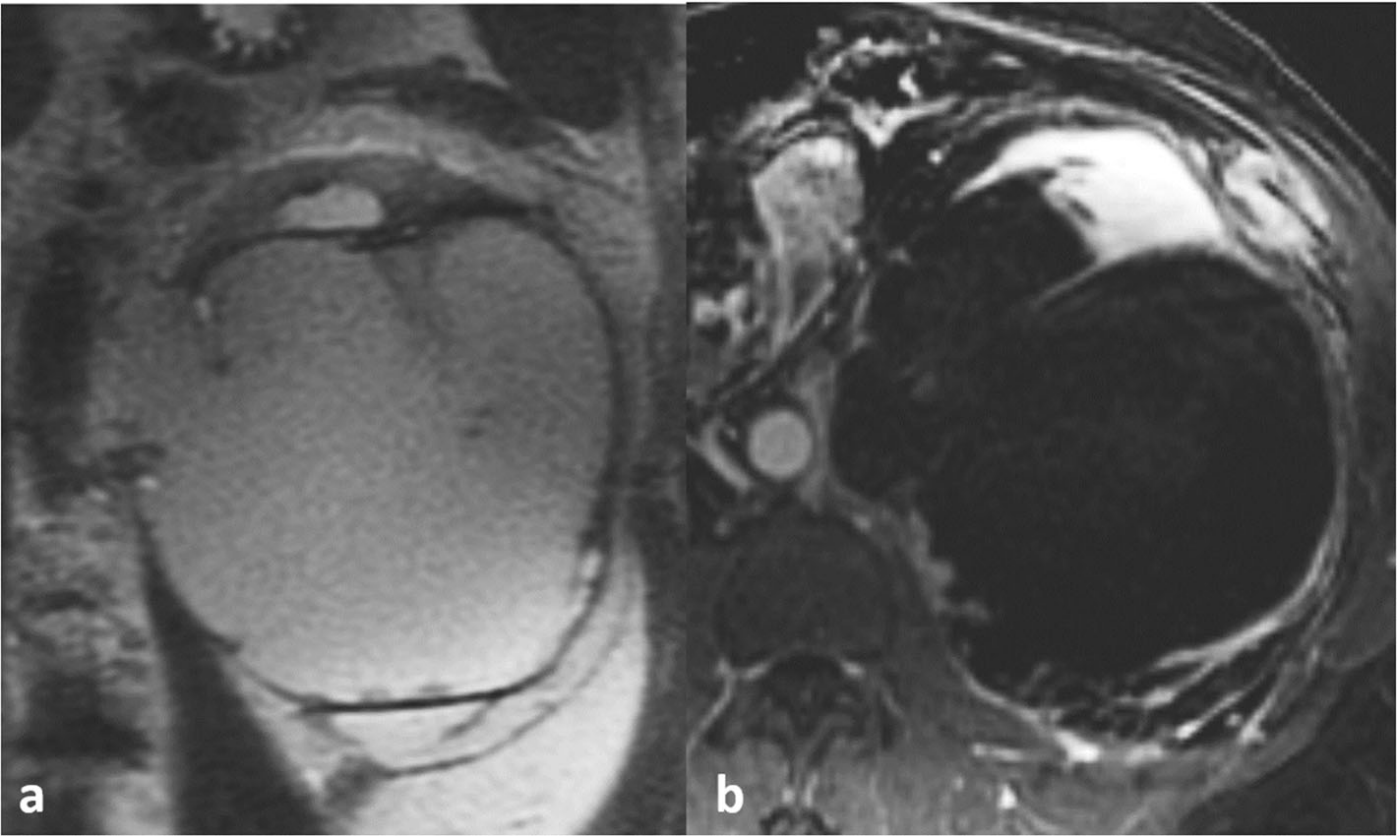
46-year-old female with fumarate hydratase-deficient renal cell carcinoma. On magnetic resonance imaging, axial T2 single-shot fast spin echo (**a**) and post-contrast 3D fat-saturated T1-weighted (**b**) sequences show a left renal mass with large cystic component

Imaging findings are summarized in Table 2. Tumors ranged in size from 2.2 cm to 18 cm (mean 10.3 cm, median 9.8 cm) and had an infiltrative rather than circumscribed margin in the majority of cases (14/16, 88 %) (Fig. 1). Regarding general tumor features, a large cystic tumor component involving greater than 75 % of the tumor was seen in 8/16 (50 %) of tumors (Fig. 2). Tumor invading the renal sinus fat was seen in 13/16 (81 %) of tumors, hilar collecting system invasion in 8/16 (50 %) (Fig. 1), and renal vein tumor thrombus in 6/16 (38 %).

**Table 2:** Imaging Features of Fumarate Hydratase-Deficient Renal Cell Carcinoma

Imaging Features	n (%)
Tumor Location and Size	
Left	8 (50)
Right	7 (44)
Bilateral	1 (6)
Size mean centimeters (range)	10.3 (2.2–18)
Size median centimeters	9.8
General Tumor Features	
Unifocal	15 (94)
Multifocal bilateral	1 (6)
Infiltrative margin	14 (88)
Circumscribed margin	2 (12)
Large cystic or necrotic component	8 (50)
Involvement of renal sinus fat	13 (81)
Involvement of hilar collecting system	8 (50)
Heterogenous enhancement	16 (100)
Peritumoral vascularity	9 (56)
Renal vein thrombus	6 (38)
Retroperitoneal nodal metastases	11 (69)
Distant metastases at presentation	12 (75)
No metastases at presentation	4 (25)
CT-Specific Tumor Features	
Calcifications	0
MRI-Specific Tumor Features	
T1 hemorrhage	7 (58)
Intravoxel fat	0
T2 signal heterogeneity	12 (100)
Diffusion restriction in solid component	11 (100)
PET-Specific Tumor Features	
FDG avidity above liver background	3 (100)
SUV_max_ mean (range)	16.4 (9.6–21.9)

*CT* computed tomography, *MRI* magnetic resonance imaging, *DWI* diffusion-weighted imaging, *FDG PET* fluorodeoxyglucose positron emission tomography, *SUV*_max_ maximum standardized uptake value

**Fig. 3:**
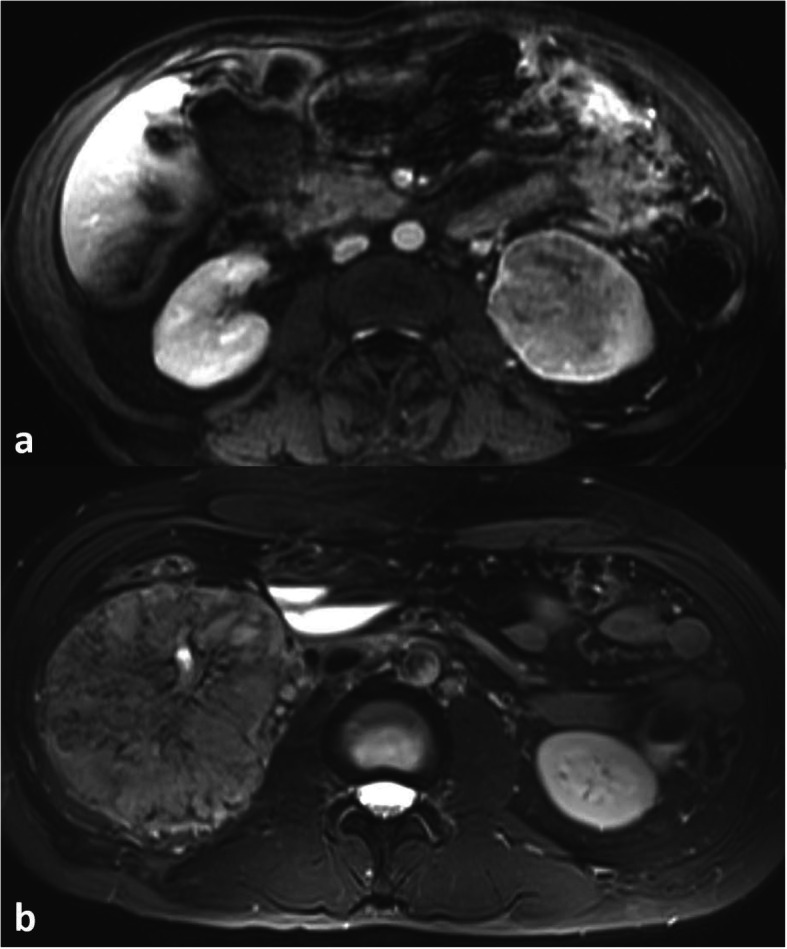
38-year-old male with fumarate hydratase-deficient renal cell carcinoma (FH-RCC). On magnetic resonance imaging, axial post-contrast 3D fat-saturated T1-weighted sequence showing a left renal mass with heterogenous enhancement (**a**). 63-year-old male with FH-RCC, axial fat-suppressed T2-weighted sequence showing a right renal mass with heterogenous T2 signal (**b**)

**Fig. 4:**
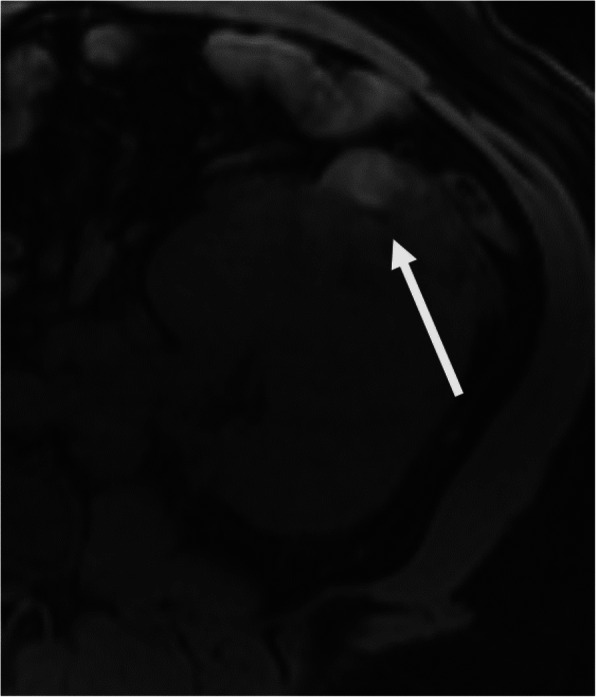
54-year-old male with fumarate hydratase-deficient renal cell carcinoma. On magnetic resonance imaging, axial non-contrast 3D fat-saturated T1-weighted sequence shows focal T1 hyperintense hemorrhage in the anterior aspect of the left renal mass (arrow)

**Fig. 5:**
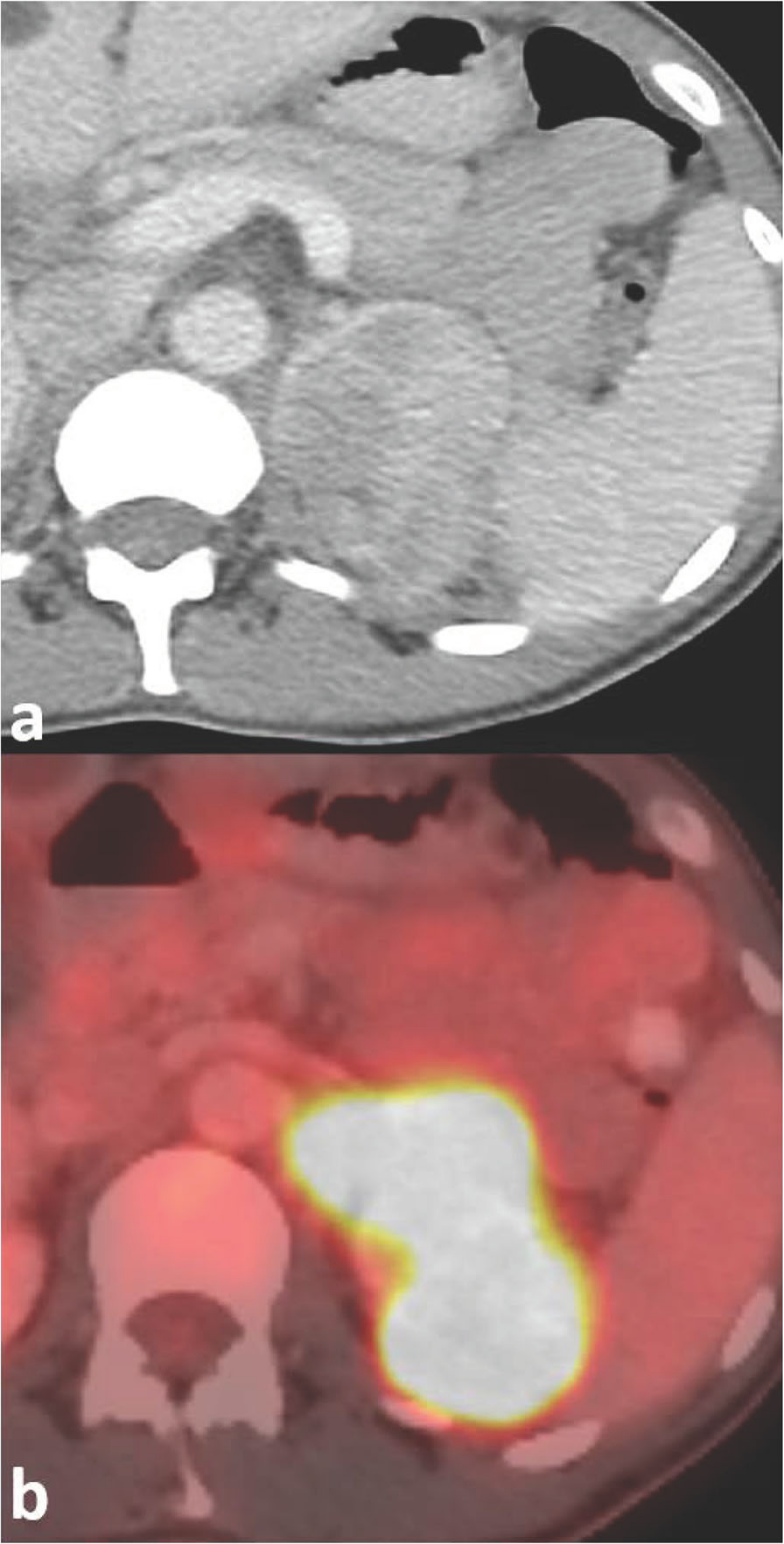
52-year-old male with metastatic fumarate hydratase-deficient renal cell carcinoma. Axial post-contrast computed tomography (**a**) and axial fused fluorodeoxyglucose (FDG) positron emission tomography/computed tomography (**b**) show an FDG-avid left renal mass (SUV_max_ 21.9)

**Fig. 6:**
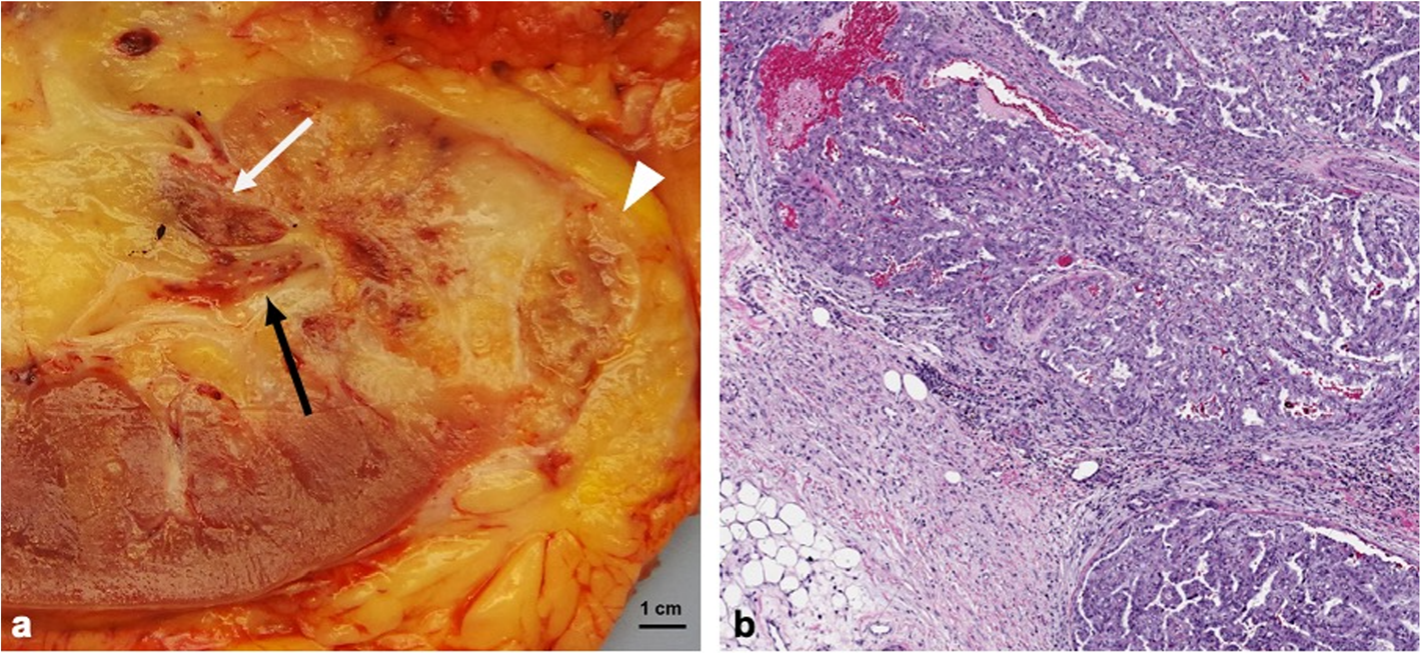
Gross and histology images from a fumarate hydratase-deficient renal cell carcinoma surgical specimen. Gross image (**a**) shows a renal mass with infiltrative margin (arrowhead), renal pelvis invasion (black arrow) and renal vein invasion (white arrow). Histology image (**b**) shows tubulopapillary growth of neoplastic cells with high grade nuclear features, invading the renal sinus fat

Regarding modality-specific features, all patients had heterogenous tumor enhancement on MRI and CT, and heterogenous T2 signal on MRI (Fig. 3). Of those patients who had diffusion-weighted imaging available for review (*n* = 11), all had diffusion restriction in the solid portions of the tumor. On MRI, T1 hyperintense hemorrhage was present in 7/12 (58 %) of cases (Fig. 4). No tumors had intravoxel fat on MRI. On CT, no tumors had calcifications. Of the three patients who had an FDG PET/CT, all had high metabolic activity (mean SUV_max_ 16.4, range 9.6–21.9), as shown in Fig. 5. An example of the gross and histologic features of one of the renal tumors is shown in Fig. 6.

At presentation, 11/16 (69 %) of patients had retroperitoneal nodal metastases and 12/16 (75 %) had distant metastases. Locations of distant metastases were the lung (*n* = 8), bone (*n* = 5), thoracic lymph nodes (*n* = 4), peritoneum (*n* = 1), pleura (*n* = 1), adrenal gland (*n* = 1), and liver (*n* = 1). Of the four patients who did not have metastatic disease at presentation, three (75 %) developed metastases within four years of diagnosis. Within a mean follow-up period of 26 months (range 9 to 60 months), 9/16 patients (56 %) died of disease. Only one patient did not have metastatic disease at time of writing, 4.5 years since diagnosis.

## Discussion

FH-RCC is a rare subtype of RCC, and to our knowledge, this is the largest single case series to describe the imaging features of FH-RCC. Regarding the clinical features of FH-RCC, this case series confirms the findings described in several larger series in the pathology literature [8, 12–14] indicating that FH-RCC usually presents at an advanced stage and in a younger population than non-FH deficient RCC. In comparison with national United States Surveillance, Epidemiology, and End Results (SEER) for tumors of the kidney and renal pelvis, this case series had a median age of 46 years at diagnosis compared with 64 years, and 75 % with distant metastases at presentation compared with 16 % [15]. One of the largest series in the pathology literature presented by Merino et al. (38 patients with 40 tumors) reported histological findings that are similar to the imaging findings presented here. Their series had similar tumor sizes, ranging from 2.3 cm to 20 cm, which were predominantly unilateral and solitary with no predilection for laterality, and showed frequent capsular and perinephric fat invasion, frequent renal vein, and inferior vena cava invasion. They showed that 21/40 tumors in their study had at least a minor cystic component, compared to 50 % of cases having a large (> 75 % of tumor volume) cystic component in this radiology case series [8].

The CT and MRI features of FH-RCC tumors in this case series appeared aggressive in most cases, illustrated by the presence of infiltrative rather than circumscribed margins, heterogeneity of MRI signal and enhancement, as well as renal sinus fat invasion. There were, however, no specific or unique features that would potentially allow them to be distinguished preoperatively from other non-FH deficient RCC on CT and MRI.

The three cases with FDG PET imaging all showed high metabolic activity within the renal mass. This feature is underpinned by tumor biology, as the FH-deficient cells have lost their ability to perform oxidative phosphorylation through the Krebs cycle, therefore depending on glycolysis, which is the basis of the Warburg effect in FDG PET imaging. There may therefore be a role for FDG PET/CT in the staging and assessment of treatment response in FH-RCC, and perhaps also in screening of asymptomatic patients with HLRCC. In contrast, typical non-FH deficient subtypes of RCC, such as clear cell RCC, are not usual considered Warburg tumors, and FDG PET has not been recommended as a routine imaging tool for RCC in professional practice guidelines [16, 17].

This study has limitations. It is a retrospective series of a small study population, and imaging with CT, MRI, and PET/CT was not available in all patients. While pathologists are becoming more aware of this entity, FH deficiency is not always tested for on IHC, and so the true scope of the imaging appearance of FH-RCC is unknown. Indeed, there is also a wider spectrum of pathologic findings in FH-RCC than previously thought, including a recently described low grade variant [18].

## Conclusions

This case series documents the appearance of FH-RCC on CT, MRI, and FDG PET/CT. On CT and MRI, the majority of these masses had invasive features and could foreseeably appear similar to any advanced stage renal tumor, although there was no direct comparison between FH-RCC and non-FH deficient RCC made in this study. The atypical finding of high metabolic activity in FDG PET imaging needs to be explored further in larger cohorts, to determine whether this could be a way to differentiate FH-deficient RCC from non FH-deficient RCC.

## Data Availability

The datasets used and/or analyzed during the current study are available from the corresponding author on reasonable request.

## Abbreviations

2SC: 2-succino-cysteine
CT: Computed Tomography
DICOM: Digital Imaging and Communications in Medicine
FH: Fumarate hydratase
FH-RCC: Fumarate hydratase-deficient renal cell carcinoma
FDG PET: Fluorodeoxyglucose positron emission tomography
HLRCC: Hereditary leiomyomatosis and renal cell carcinoma
IHC: Immunohistochemical
MRI: Magnetic Resonance Imaging
PACS: Picture Archive and Communication System
SEER: Surveillance, Epidemiology, and End Results
SUVmax: Maximum standardized uptake value
